# Ending tuberculosis in Europe - resetting the course in the post-COVID-19 era

**DOI:** 10.2807/1560-7917.ES.2023.28.12.2300164

**Published:** 2023-03-23

**Authors:** Andrea Ammon, Hans Kluge

**Affiliations:** 1 European Centre for Disease Prevention and Control (ECDC), Stockholm, Sweden; 2 World Health Organization Regional Office for Europe (WHO/Europe), Copenhagen, Denmark

**Keywords:** tuberculosis, Europe

On 24 March 1882, Robert Koch announced his discovery of *Mycobacterium tuberculosis* as the causal agent of tuberculosis (TB) [1]. For centuries, the disease has claimed hundreds of thousands of lives, bringing economic hardship to patients, their families and health systems. Looking back from where we stand today, on 24 March 2023, World TB Day, we have made great progress in tackling this preventable and curable disease, including progress on the social and economic factors linked to TB.

In 2015, European countries endorsed target 3.3 of the Sustainable Development Goals (SDG) that aims at ending the TB epidemic by 2030 [2]. To support this global vision, the End TB Strategy defined three main targets for reducing the TB disease burden (Table) [3].

**Table:** Global and World Health Organization European Region tuberculosis targets [3,12]

Roadmap (year)	Indicator	Milestones	Target
Sustainable Development Goals - target 3.3 (2015)	TB incidence per 100,000 population	NA	By 2030, end epidemics of AIDS, TB, malaria and neglected tropical diseases and combat hepatitis, waterborne diseases and other communicable diseases
End TB Strategy (2015)	Reduction in the TB incidence per 100,000 population compared with the 2015 baseline	20% reduction by 2020; 50% reduction by 2025	80% reduction by 2030
	Reduction in the number of TB deaths compared with the 2015 baseline	35% reduction by 2020; 75% reduction by 2025	90% reduction by 2030
	TB-affected households face catastrophic costs^a^	NA	Zero households by 2020
United Nations high-level meeting on TB (2018–2022)	Number of people treated for TB between 2018 and 2022	Progress achieved by 2020	40 million people treated for TB, including: - 3.5 million children - 1.5 million people with drug resistant TB, including 115,000 children
	Number of people provided with TB preventive treatment between 2018 and 2022	Progress achieved by 2020	At least 30 million people provided with TB preventive treatment, including: - 6 million people living with HIV - 4 million children under 5 years of age, and 20 million people in other age groups who are household contacts of people affected by TB
	Funding for universal access to TB prevention, diagnosis, treatment and care	Progress achieved by 2020	At least $13 billion annually by 2022
	Funding for TB research	Progress achieved by 2020	At least $ 2 billion annually from 2018 to 2022
TB Action Plan for the WHO European Region 2023–2030	Reduction in the TB incidence rate per 100,000 population compared with 2015 baseline	50% reduction by 2025	80% reduction by 2030
	Reduction in the number of TB deaths compared with 2015 baseline	75% reduction by 2025	90% reduction by 2030
	Treatment success rate among MDR/RR-TB	80% by 2025	85% by 2030

AIDS: acquired immunodeficiency syndrome; HIV: human immunodeficiency virus; RR: rifampicin-resistant; MDR: multidrug-resistant; NA: not applicable; TB: tuberculosis; WHO: World Health Organization.

^a^ Catastrophic costs due to TB are defined as the total costs (i.e., the sum of direct medical expenditures, direct nonmedical expenditures and indirect costs), paid out-of-pocket by people receiving TB treatment, that exceed 20% of their household’s annual income [5].

Three years later, in September 2018, global leaders at the first United Nations (UN) General Assembly High-Level Meeting on the Fight Against TB agreed on ambitious targets to be achieved by 2022. These include increased access to TB prevention and treatment and increased funding for both TB services and TB research, to accelerate progress towards the 2030 goal [4].

But since then, progress to end TB has stalled, even reversed, with geographically uneven performance across the World Health Organization (WHO) European Region. While most countries in Western Europe appear to be on track for TB elimination, Eastern European and Central Asian countries continue to experience a high burden of drug-resistant (DR) TB [5]. TB incidence in the European Union/European Economic Area (EU/EEA) is low, with 24 of 30 countries reporting less than 10 cases per 100,000 population in 2021, according to the latest TB surveillance report from Europe [6]. These countries are encouraged to maintain the low rate already achieved, to go beyond the 2030 global target and to strive to reach the pre-elimination phase, defined as < 10 notified TB cases per million population per year [7]. In this issue of *Eurosurveillance*, Cristea et al. present their analysis of the progress achieved between 2018 and 2021 in the EU/EEA towards the 2030 targets for TB elimination [8]. Their results show the continued reduction in TB notification in the EU/EEA, but also highlight the challenges faced by low-incidence countries, such as the low treatment success rates among people with DR-TB [8].

Worryingly, 22% of the global cases of multidrug-resistant or rifampicin-resistant TB (MDR/RR-TB) [6] and 42% of pre-extensively DR-TB live in the European Region [5]. One in three pulmonary TB patients in Europe has rifampicin-resistant TB, and of these 30% have resistance to fluoroquinolones [6]. In addition, the treatment success for MDR/RR-TB at 57%, remains way below the regional target of 85% [5]. TB-HIV coinfection is on the rise, with an estimated HIV infection rate of 13% among incident TB cases in 2021, compared with 9.7% in 2015 [5].

There are multiple reasons why targets set in 2015 and then in 2018 have not been achieved. Consecutive crises such as natural disasters, conflicts, and the COVID-19 pandemic have severely impacted health systems, and jeopardised gains made, including the control of MDR/RR-TB and TB-HIV co-infection. Fragmented approaches and working in silos rather than collaborating and integrating efforts and resources, have slowed down success in Europe and globally.

Just 3 years ago, the WHO European Region was on a good trajectory, with the fastest decline in TB incidence and mortality globally [5]. By 2020, it had surpassed the End TB strategy milestone of 20% cumulative reduction for TB incidence compared with the 2015 baseline [5]. However, the COVID-19 pandemic has derailed progress towards ending TB, along with many other health priorities, and continues to have damaging impact on the burden of TB. It caused service disruptions and barriers to care, resulting in a substantial reduction in the number of TB patients being diagnosed and in the number of patients enrolled in treatment [9,10]. This will lead to an increase in TB deaths and greater transmission of infection, followed with some lag-time, by an increase in numbers of people developing TB [5].

The ongoing war in Ukraine has caused an additional setback, triggering an escalating humanitarian crisis in Europe with direct and indirect impacts on people’s lives and health, as well as on Ukraine’s health system. As a consequence, there is a risk of increased HIV and MDR/RR-TB infection rates in Ukraine and in Europe more widely, due to limited access to diagnosis and treatment and to population movement [11,12]. Guthmann et al., also in this issue, present an experience of an active screening strategy to detect active TB cases among Ukrainian refugees soon after arrival to France [13]. Their study discusses barriers to access TB care that must be tackled as part of a comprehensive package of cross-border interventions to guarantee continuity of care for people with TB [13,14]. WHO and ECDC recommend targeted screening of Ukrainian refugees belonging to certain groups at risk of TB, such as people living with HIV or those who are contacts of TB patients, instead of universal screening [15].

The economic impact of the war in Ukraine across Europe may affect governments’ abilities to fund the commitment needed to scale up the TB response.

The challenges are enormous, and they demand a determined response. To get back on track, we need revitalised and urgent political and financial commitment, and an agile and adapted response to TB. We also need to handle new challenges and threats proactively. For example, the article by Martínez-Lirola et al. in this issue reminds us that TB must also be considered within the One Health approach; by using whole genome sequencing the authors uncovered hidden transmission routes for TB in Spain [16].

In our favour, we have several new tools to support prevention and control of TB and DR-TB in Europe. For the first time, the duration of MDR/RR-TB treatment is 6 months – the same as for drug-susceptible TB, shortened from treatment of 18 months or longer; an important breakthrough in TB response. Further, these shorter, injection-free treatment regimens for MDR/RR-TB will substantially increase coverage and treatment success [17], while new rapid molecular diagnostics, digital health solutions and innovative service-delivery approaches are reshaping the TB response.

To truly end TB, we must interrupt transmission by finding people with active TB before they infect others and prevent those already infected from developing the disease. TB-preventive treatment (TPT) remains the main public health intervention and top priority for elimination, particularly for population groups at high risk of infection and progression from infection to disease, including people living with HIV and/or in contact with pulmonary TB patients [18]. Providing TPT to 450,000 people in the European Region between 2023 and 2030 [19], will require a massive scale-up of efforts. It also requires an investment in revised approaches to targeted systematic screening for TB disease and testing for TB infection, better linkages between services and greater integration with primary healthcare. Unfortunately, European countries – particularly EU/EEA countries – currently have limited availability of TB medicines recommended in the new and shorter TPT regimens and the novel treatment options for MDR/RR-TB. This limited access is caused by lack of registration of rifapentine, an essential drug in the new TPT and shorter treatment for drug-susceptible TB, on the European market [20]. What jeopardises further progress in ending TB in Europe is the high market cost of core medicines for DR-TB treatment [21].

In September 2022 there was a renewed political commitment for ending TB in Europe when countries endorsed the 2023–2030 TB Action Plan [22]. This regional plan is fully aligned with the SDGs and End TB Strategy and aims at *“ending the spread of drug-susceptible and drug-resistant TB by achieving universal access to prevention, diagnosis and treatment in all Member States of the Region”* [12].

The year 2023 provides another opportunity to renew global TB commitments. On 22 September, during the UN General Assembly, world leaders will gather in New York to participate in a follow-up high-level meeting on TB. The theme of the meeting is *Advancing science, finance and innovation, and their benefits, to urgently end the global tuberculosis epidemic, in particular by ensuring equitable access to prevention, testing, treatment and care* [23]. At the meeting, global leaders will commit to new targets and set new milestones to end TB. This should not be an empty exercise. Rather, these new commitments must lead to action and accountability at all levels. The meeting will be co-facilitated by Poland and Uzbekistan, both countries in the WHO European Region, providing an opportunity to step up regional leadership. We call upon European countries across the socioeconomic spectrum, represented by heads of state and health leaders alike, to rise to the challenge. The commitments should be a promise we make to help people who are most in need – as TB is still largely a disease of poverty and neglect, shrouded in stigma and discrimination, affecting some of the most vulnerable people.

For our part, the European Centre for Disease Prevention and Control and the WHO Regional Office for Europe, are committed to supporting all efforts and work towards ending TB in Europe by 2030 and beyond.

## Note

Andrea Ammon is the Director of the European Centre for Disease Prevention and Control (ECDC) and Hans Kluge is the World Health Organization (WHO) Regional Director for Europe.
